# Limiting Albuterol Use by EMS at the Start of the COVID-19 Pandemic: A Retrospective Analysis of Rapid Deimplementation

**DOI:** 10.5811/westjem.47030

**Published:** 2025-11-18

**Authors:** Renoj Varughese, Susan J. Burnett, Hilary Kirk, Ian Wallis, Nan Nan, Chang-Xing Ma, David Hostler, Brian M. Clemency

**Affiliations:** * University of Buffalo, The State University of New York, Jacobs School of Medicine and Biomedical Sciences, Department of Emergency Medicine, Buffalo, New York; † University of Buffalo, The State University of New York, School of Public Health and Health and Health Professions, Department of Exercise and Nutrition Sciences, Buffalo, New York; ‡ University of Buffalo, The State University of New York, School of Public Health and Health and Health Professions, Department of Biostatistics, Buffalo, New York

## Abstract

**Introduction:**

Deimplementation is the process through which an existing practice, procedure, or protocol is discontinued. Past deimplementation efforts in emergency medical services (EMS), such as reduction of liberal oxygen administration, backboard use, and lights and sirens responses, have been slow in rates of change and had varying levels of adoption. Our objective in this study was to analyze the deimplementation of albuterol administration in the beginning of the 2019 novel coronavirus (COVID-19) pandemic for the adoption of deimplementation guidelines, rate of change, and factors leading to this change in EMS practice.

**Methods:**

Using the 2020 National Emergency Medical Services Information System (NEMSIS) dataset, we analyzed the change in EMS calls with albuterol administration following the US Centers for Disease Control and Prevention (CDC) advisory recommending limiting aerosol-generating procedures in response to the COVID-19 pandemic.

**Results:**

The 2020 NEMSIS dataset included 43,488,767 total records, and 449,290 (1.0%) records included at least one albuterol administration. Calls with albuterol administration dropped 61.7% in a near-linear fashion in the six weeks following the publication of the CDC’s guidance (from March 8–April 18, 10,426 absolute reduction; from 16,891 to 6,465, in average calls per week with albuterol administration). In the period before the guidance, there were on average 16,891 calls with albuterol administration of 640,597 (2.6%) calls per week. In the period after the guidance, there were, on average, 6,465 calls with albuterol administration of 601,943 (1.1%) calls per week. Therefore, while total EMS calls declined by 6% during the transition period, the proportion of albuterol calls within this decline went down by 1.5% (2.6% to 1.1%), reflecting rapid deimplementation.

**Conclusion:**

Deimplementation of albuterol administration in the beginning of the COVID-19 pandemic was significant in its rate and success in adherence to guidelines when compared to other changes in EMS policies, procedures, and protocols. A better understanding of deimplementation can guide future EMS efforts to phase out ineffective practices while minimizing disruption to care.

## INTRODUCTION

Deimplementation is the process through which an existing practice, procedure, or protocol is discontinued, reduced, reversed, or replaced.^1^ While some consider deimplementation to be the opposite of implementation, differences in the motivations, procedures, and outcomes make the process more nuanced than a simple mirroring might suggest.^1^,^2^ Adjustments and improvements in emergency medical services (EMS) practices are common, but the rate of change for discontinuation of existing practices is often slow or gradual.^3^,^4^ One of the most notable deimplemented EMS procedures in recent history was the transition from the long-standing spinal immobilization paradigm.^5^ A key feature of this deimplementation was a major reduction in the use of the long spine board.^6^,^7^ A database review of over 25,000 electronic patient care records over a 10-year period demonstrated a considerable decrease in immobilization practices after a spinal motion restriction protocol was implemented, but there was an eventual plateau in the rate of use, and application of the protocol was inconsistent.^8^

American Heart Association (AHA) guideline changes have also led to changes in established practices. In 2010, recommendations for liberal oxygen administration were changed to titration to blood oxygen saturation and symptoms in patients with acute coronary syndrome.^9^ In an evaluation of paramedic student documentation in the two years following the protocol change, decrease in oxygen administration was significant, but half of the patients who received the treatment still did not meet AHA criteria for the intervention.^10^ Furthermore, among the Resuscitation Outcomes Consortium sites, it took on average over 13 months to implement the 2005 AHA Guidelines for Cardiopulmonary Resuscitation and Emergency Cardiac Care.^4^

Finally, data over the past several decades indicate clear correlations between use of lights and sirens (L&S) in EMS and injuries or death of EMS professionals, their patients, and members of the public.^11^,^12^ Revelations about the lack of time saved or improved patient outcomes in most cases have not reduced L&S use in a majority of EMS responses and transports.^11^,^13^ Emergency medical services professionals understand the reasons for deimplementation of L&S, yet they continue to use them.^14^ Public expectations of EMS responses and the need for EMS professionals to manage them may be associated with the slow decrease in L&S use.^13^

On January 20, 2020, the US Centers for Disease Control and Prevention (CDC) confirmed the first case of 2019 novel coronavirus (COVID-19) in the United States.^15^ The unknown nature of the disease presented unique challenges for EMS clinicians. There was concern that being in enclosed spaces with patients suspected of having COVID-19 for an extended period could increase the risk of EMS clinicians contracting the virus. On March 10, 2020, the CDC published guidance and recommendations for EMS clinicians and organizations, which included a cautionary advisory regarding aerosol-generating procedures (AGP), such as nebulizing medications.^16^ Nebulized albuterol has been a standard treatment for EMS clinicians treating patients with acute bronchospasm for decades. Our goal in this study was to describe the rapid deimplementation of prehospital albuterol administration at the start of the COVID-19 pandemic.

**Population Health Research Capsule**

What do we already know about this issue?

*Adjustments and improvements in EMS practices are common, but the rate of change for discontinuation of existing practices is often slow.*

What was the research question?

*How did EMS albuterol use change after the US Centers for Disease Control and Prevention (CDC) recommended limiting aerosol-generating procedures?*

What was the major finding of the study?

*The proportion of albuterol calls went down from 2.6% to 1.1% (P < .001) after the CDC recommendations.*

How does this improve population health?

*A better understanding of deimplementation can guide future EMS efforts to phase out ineffective practices while minimizing disruption to care.*

## METHODS

We performed a retrospective review of data from the 2020 National Emergency Medical Services Information System (NEMSIS) public-release dataset, which was released in May 2021. We used the NEMSIS data dictionary v3.5.0, which was released in 2019 and was the available data dictionary for 2020. This dataset has been previously described and used for COVID 19-related research.^17^–^20^ This study was designated not human subjects research by the University at Buffalo Institutional Review Board (00005516). The abstractors were not blinded to the study hypothesis.

We identified EMS encounters that occurred in 2020, with at least one albuterol administration. Specifically, the data field, *eMedications.03*, was searched for albuterol and/or albuterol with ipratropium. Next, we identified the date of the call using a two-step process. First, the dispatch date and time were identified using the *eTimes.01* field. If the dispatch date and time were not recorded, then the date and time of the medication administration were identified using the *eMedications.01* field. If there was no accompanying date or time in either field, we excluded the record. Dates were sorted by the week in which they occurred. We used weekly intervals, in place of daily or monthly intervals, to reduce the impact of outliers while allowing for timelier identification of change. Encounters that occurred during partial weeks at the start and end of the year were excluded. We calculated the proportions as number of calls per week with albuterol administration divided by number of calls dispatched in a given week.

The weeks prior to the publication of the March CDC guidance document were designated as the before period. The weeks between the CDC document’s publication date and the nadir of albuterol administration were designated as the transition period. The nadir was defined as the week with the lowest number of calls with albuterol administrations. The weeks following the nadir were designated as the after period. We performed the descriptive statistic calculations using SAS 9.4 (SAS Institute Inc, Cary, NC).

## RESULTS

The complete 2020 NEMSIS dataset included 43,488,767 total records; of those records, 449,290 (1%) reported at least one albuterol administration. We excluded 12,001,540 cases because of the lack of a valid date in the record. We also excluded encounters from the incomplete weeks at the beginning and the end of the study period. The study time frame was January 5–December 26, 2020. Our analysis included a total of 30,726,473 records, 431,939 of which included documentation of at least one albuterol administration.

The CDC guidance was released on March 10, 2020 (week 11), and stayed in effect through the remainder of the study period. The before period was January 5, 2020 (week 2) to March 7, 2020 (week 10), during which there was an average of 16,891 calls (2.6% of all calls) with albuterol administration per week. The transition period was March 8, 2020 (week 11) to April 18, 2020 (week 16). Calls with albuterol administration fell in a near-linear fashion during this period. The after period was from April 19, 2020 (week 17) to December 26, 2020 (week 52), during which there was an average of 6,465 calls (1.1% of all calls) with albuterol administrations per week (Table 1).

**Figure 1:**
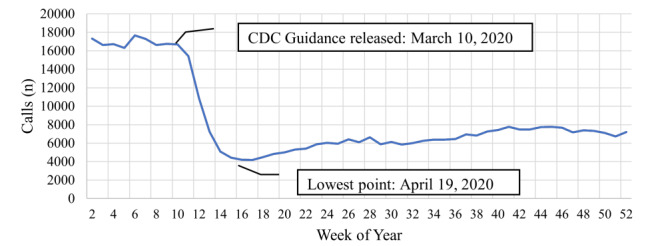
Number of emergency medical services calls with albuterol administration throughout 2020 in a study of deimplementation of prehospital administration of nebulized albuterol at the beginning of the COVID-19 pandemic (by week). *CDC*, Centers for Disease Control and Prevention.

**Figure 2:**
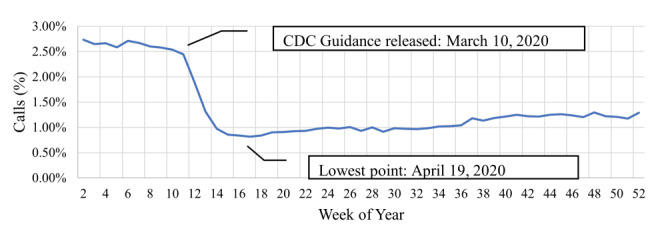
Proportion of the EMS* calls that had prehospital albuterol administration over all calls through 2020 in a study of deimplementation of prehospital administration of nebulized albuterol at the beginning of the COVID-19 pandemic (by week). This included a total of 30,726,473 records, 431,939 of which included documentation of at least one albuterol administration. *CDC*, Centers for Disease Control and Prevention; *EMS*, emergency medical services.

This represents a 6% reduction in overall call volume between the before and after periods. The number of EMS calls with albuterol administrations in the after period compared to the before period was statistically significant (*P* < .001). There was a 61.7% reduction in the average albuterol administration between the before and after periods. All calls with albuterol administrations were grouped by week and graphed over the year (Figure 1). The proportion of all calls with albuterol administrations to the total number of calls were grouped by week and graphed over the year (Figure 2).

## DISCUSSION

The deimplementation of albuterol administration shortly after the release of the CDC’s guidance is unlike prior EMS deimplementation efforts because of its speed and widespread adherence. Deimplementation of AGP use was unique since it was not a reversal or replacement of a defunct or disproven practice, nor was the recommendation based on a preponderance of evidence of the practice’s danger or obsolescence.^1^,^2^

This precipitous and successful deimplementation of nebulized medications may have been driven by the prescribed action of ceasing AGPs, the larger sociocultural context of the pandemic, the healthcare system context, or the responsibility placed on EMS clinicians to change their patient care practices. Factors associated with deimplementation delays or efficacy may be related to systemic or logistic issues, such as lack of resources to conduct education or difficulty in coordinating with healthcare partners.^4^,^21^,^23^

## LIMITATIONS

This study examined the fixed elements in the NEMSIS dataset. A date/time in e.Times01 is not mandatory, and the date/time field in *e.Medications.01* is nillable, which contributed to the exclusion of 27% of the records from the study. It did not include patient factors and information available in the narrative section of EMS records, nor did this study review patient outcomes. The overall drop in total patient call volume may have contributed to a decrease in patients for whom AGPs would be typically indicated. This study did not analyze the routes of albuterol administration, recorded in the NEMSIS field *eMedications.04*, as the variability in medication route-labeling practice patterns would have added another confounding factor. Future research could study the EMS clinicians’ perspectives on the deimplementation of AGP use. Finally, this methodology did not account for potential seasonal variations in respiratory illnesses.

## CONCLUSION

Deimplementation efforts in EMS are traditionally slow or gradual over time. In the case of the use of aerosol-generating procedures in the beginning of the COVID-19 pandemic, deimplementation occurred in the six weeks after CDC guidelines were published.

## Figures and Tables

**Table 1:** Number of emergency medical services (EMS) calls and EMS calls with albuterol administration throughout 2020 in a study of deimplementation of prehospital administration of nebulized albuterol at the beginning of the COVID-19 pandemic.

	Before Transition	Transition	After Transition
Time period	Weeks 2–10 (January 5 – March 7)	Weeks 11–16 (March 8 – April 18)	Weeks 17–52 (April 19 – December 26)
Total calls in the time period	5,765,374	3,291,118	21,669,981
Total calls with albuterol administration in the time period	152,018	47,177	232,744
Average total calls per week (Minimum – Maximum)	640,597 (626,675 – 656,833)	548,520 (499,148 – 631,619)	601,943 (514,914 – 662,756)
Average calls per week with albuterol administration (Minimum – Maximum)	16,891 (16,320 to 17,332)	7,863 (4,190 – 15,428)	6,465 (4,186 to 7,766)
Percentage of total calls with albuterol administration	2.6%	1.4%	1.1%
